# An Irritable Infant and the Runaway Redback: An Instructive Case

**DOI:** 10.1155/2011/125740

**Published:** 2011-09-28

**Authors:** Thomas R. Ward, James A. Falconer, John A. Craven

**Affiliations:** Canberra Hospital, P.O. Box 11, Woden, ACT 2606, Australia

## Abstract

The envenomation syndrome of Redback spider bites, lactrodectism, is distinctive. However diagnosis can be difficult due to an atypical presentation. We describe the case of a 1 year old boy with irritability, diaphoresis and reduced oral intake, in whom a diagnosis was made of redback spider bite. Successful resolution of symptoms was achieved following treatment with antivenom. The symptoms and management of redback spider bites is discussed.

## 1. Case History

A one-year-old boy was referred to the emergency department by his family's general practitioner after sudden onset of irritability, reduced oral intake, and diaphoresis 24 hours prior to presentation. The general practitioner reported a tense abdomen and was worried about appendicitis or intussusception. There was no vomiting, diarrhea, coughing, or rhinorrhea. His mother reported a transient macular erythematous rash on his trunk and shoulders which disappeared after approximately one hour. 

**Figure 1:**
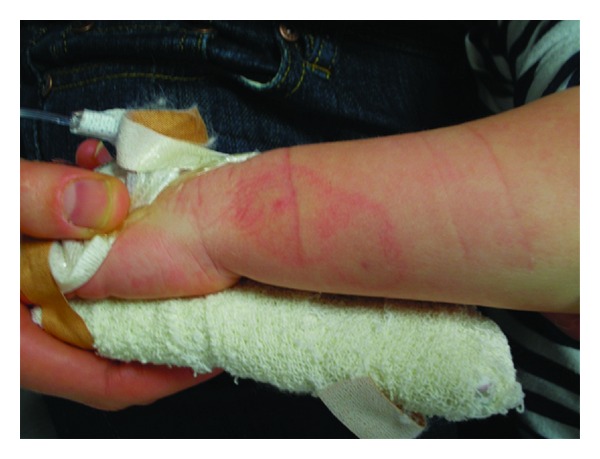
Bite marks on patient's skin.

On examination the child was irritable and diaphoretic, but haemodynamically stable and afebrile. The abdomen was soft, and bowel sounds were present. The macular erythematous rash reappeared approximately two hours after presentation and lasted for approximately half an hour. Two small erythematous marks were evident on the child's left forearm and were surrounded by a pale region approximately 10 mm in radius with an outer erythematous annulus (Figure 1). Other examination findings were unremarkable. On further specific questioning, the mother recalled seeing a small black spider with a red stripe on its back whilst changing her son on the change table of the local pool approximately 1 hour before the onset of symptoms. The family had recently moved to Australia, so the mother was unfamiliar with local arachnids. 

Routine blood tests and blood sugar level were all unremarkable.

Based upon the clinical history and the presence of bite marks, a diagnosis of a Redback spider bite was made. Redback spider antivenom (CSL) was given intravenously (500 units in 100 mL of normal saline). The child made a full recovery within 30 minutes and was discharged home three hours later.

## 2. Discussion

**Figure 2:**
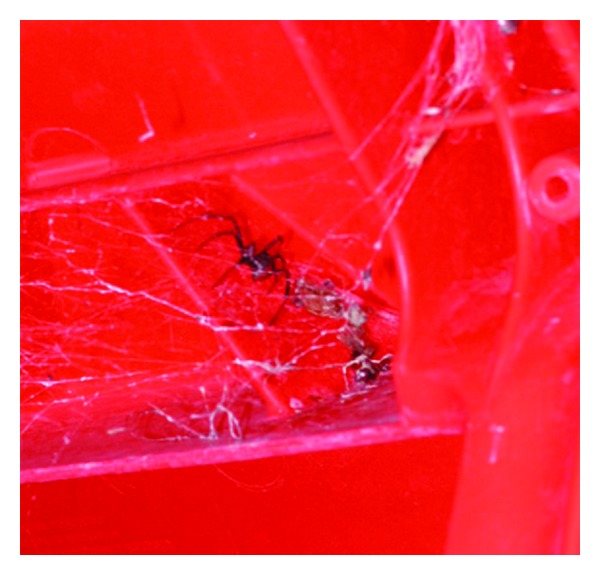
A Redback spider in its native habitat. Photo taken by author (JAC) under a chair in the Alice Springs Hospital cafeteria, 2002.

Redback spiders (*Latrodectus hasselti*) not only are now prevalent throughout Australia (Figure 2) but have been reported in parts of Japan and New Zealand [1]. The venom contains a neurotoxin, alpha-latrotoxin, which leads to generalized release of acetylcholine and catecholamines at nerve terminals in the autonomic nervous system [2]. This results in characteristic symptoms—latrodectism—which involves intense local or regional pain, regional or generalized diaphoresis, fever, paraesthesia, patchy paralysis, localized lymphadenopathy and hypertension [2, 3]. Children often present with a triad of irritability, hypertension and diaphoresis [4]. Pain can mimic an acute abdomen or testicular torsion. 

The median duration of symptoms is 48 hours, and almost all cases resolve within one week. Patients may not readily identify the cause as there may be a delay prior to onset of symptoms. Furthermore, evidence at the bite site, such as erythema, is not always present [2]. Ice and simple analgesics are useful first aid measures. Pressure immobilisation bandages are not recommended.

CSL Redback Spider Antivenom, an equine IgG Fab, is indicated for treatment of systemic envenomation and may also be used for local pain unresponsive to simple analgesics. It may be used as a diagnostic tool in patients with clinical symptoms of envenomation but no history of bite. The standard treatment for all patients is 1000 units (2 vials) of antivenom given as intramuscular (im) injection undiluted or intravenously (iv) diluted in 100 mls normal saline over 20 minutes. Although a further 2 vials can be given every 2 hours until symptoms fully resolve, if there is no symptomatic improvement, the diagnosis should be revisited. Due to the small size of the child in the discussed case, 500 units was administered as a trial dose, and no further treatment was required as all symptoms resolved. Symptoms completely resolve in approximately 85% of patients within 24 hours of treatment [5]. 

Antivenom can be given intravenously or intramuscularly. Randomised controlled trials have demonstrated faster resolution of pain with intravenous compared with intramuscular administration, although undiluted intravenous administration results in higher rates of adverse reactions [3, 5]. Acute hypersensitivity reactions occur in approximately 5% of subjects, with the incidence of anaphylaxis reported as 0.5%. Pretreatment with steroids or adrenaline is not recommended [5, 6]. A serum sickness may develop in up to 16% of subjects between 4 to 14 days after administration of antivenom [5]. This may manifest as fever, rash, arthralgia or myalgia. Oral prednisolone (50 mg adults, or 1 mg/kg children, for 5 days) ameliorates symptoms.

Administration of antivenom should be considered carefully as the mortality attributed to Redback spider bites is low and resolution of symptoms will eventually occur. However the level of patient distress due to symptoms is often high and systemically envenomed patients welcome the complete and often dramatic resolution of symptoms that generally occurs with treatment.

##  Key Points

- Redback spider (*Latrodectus hasselti*) bites should be considered in the list of differential diagnoses for an infant with symptoms of irritability and diaphoresis.
- The onset of symptoms may be delayed after the bite, so a careful history prior to symptom onset is important.
- Without treatment, symptoms can last for several days and can be extremely unpleasant, although rarely fatal.
- If there is a high index of suspicion, the benefits and risks of antivenom need to be considered. The main benefit is resolution in approximately 85% of cases after one day. The main risks are immediate hypersensitivity reaction or late onset of serum sickness.
